# No Association of Four Candidate Genetic Variants in MnSOD and SYNIII with Parkinson's Disease in Two Chinese Populations

**DOI:** 10.1371/journal.pone.0088050

**Published:** 2014-02-26

**Authors:** Wen Juan Yu, Nan Nan Li, Eng King Tan, Lan Cheng, Jin Hong Zhang, Xue Ye Mao, Xue Li Chang, Dong Mei Zhao, Qiao Liao, Rong Peng

**Affiliations:** 1 Department of Neurology, West China Hospital, Sichuan University, Chengdu, Sichuan Province, PR China; 2 Duke–NUS Graduate Medical School, Singapore, Singapore; 3 Department of Neurology, Singapore General Hospital, National Neuroscience Institute, Singapore, Singapore; 4 Department of Internal Medicine, Wangjiang Hospital, Sichuan University, Chengdu, Sichuan Province, PR China; UCL Institute of Neurology, United Kingdom

## Abstract

**Background:**

The manganese superoxide dismutase (MnSOD) gene, which encodes a chief reactive oxygen species (ROS) scavenging enzyme, has been reported to be associated with the risk of developing sporadic Parkinson's disease (PD) in some Asian races and the synapsin III (SYN3) gene with some neuropsychiatric diseases. Objective: To explore the associations between the MnSOD and SYN III variations and PD in two Chinese populations from mainland China and Singapore.

**Methods:**

We recruited 2342 subjects including 1200 sporadic PD patients and 1142 healthy controls from two independent Asian countries. Using a case-control methodology, we genotyped the single nucleotide polymorphisms (SNP) in MnSOD (rs4880) and SYN III (rs3788470, rs3827336, rs5998557) to explore the associations with risk of PD.

**Results:**

The results showed the genotype distributions and minor allele frequencies (MAF) of MnSOD (rs4880) and SYN III (rs3788470, rs3827336, rs5998557) were not significantly different between PD patients and healthy controls in mainland China and Singapore, as well as in merged populations.

**Conclusions:**

The variations of MnSOD (rs4880) and SYN III (rs3788470, rs3827336, rs5998557) were not major risk factors for PD among Chinese, at least in our study populations.

## Introduction

Parkinson’s disease (PD; OMIM #168600), a debilitating and progressive neurodegenerative disorder, is characterized by four chief symptoms (tremor, rigidity, bradykinesia, postural reflex impairment) and nonmotor (sleep disturbances, constipation, depression, autonomic dysfunction, etc.) symptoms, and affects 2% of people age 65 or older [1]. The widely accepted pathologic mechanism of PD is the selective degeneration of dopaminergic neurons (DA) in the substantia nigra pars compacta and the consequent depletion of dopamine. However, the cause of this degeneration is unclear. It is likely that a complex network of environmental factors and genetic predisposition may account for the development of PD [2].

A recent report by Fong and co-workers showed an increased risk of PD among Taiwanese C allele carriers of MnSOD with history of pesticide exposure [3]. Additionally, Shimoda have reported in 1997 that the Mn-superoxide dismutase (*MnSOD*) C allele is significantly associated with familial PD in Japanese patients [4]. However, no association with rs4880 were detected in two Genome-wide association studies and other three European studies [5]–[9]. The synapsin III (*SYN3*) gene is located close to one of the multiple sclerosis susceptibility regions (in 22q13.1) [10] and a candidate region implicated in linkage studies of schizophrenia [11]. A number of studies have reported the association of the polymorphism of *SYN3* with multiple sclerosis and several neuropsychiatric diseases [12]–[14]. However, heretofore no research about the association of *SYN3*(rs997120,rs9619283,rs2051569) and PD were reported.

Therefore, in order to test whether the previously described association between *Mn-SOD* and PD is consistent in Chinese population and try to explore the relationship between *SYN3* and PD, we performed a large case-control study including 2342 individuals from mainland China and Singapore. To our knowledge, this is the first study that has attempted to explore the association between SYN3 and PD.

## Subjects and Methods

### Subjects

The mainland China series is composed of 810 sporadic PD patients (mean age 57.83 years, range from 30 to 90, 43.3% women) and 750 neurologically normal healthy control subjects (mean age 55.25 years, range from 30 to 91, 46.9% women). All individuals were recruited from the Department of Neurology at West China Hospital. All patients were examined and followed up longitudinally by two movement disorders neurologists and diagnosed with idiopathic PD according to the UK Parkinson’s Disease Society Brain Bank Clinical Diagnostic Criteria [15]. Patients with a positive family history of PD were excluded. All control individuals (the mean age 55.25 years, range from 30 to 91) were healthy volunteers without any neurological or psychiatric diseases and were recruited from the same ethnic group, matched by age and gender. Written informed consent was obtained from all participants. This study was approved by the Ethics Committee of Sichuan University.

The Singapore series is composed of 390 sporadic PD patients (mean age 67.12 years, range 38 to 91, 42.8% women) and 392 age-, gender- and ethnicity-matched neurologically healthy controls (the mean age 58.87 years, range 47 to 89, 46.4% women). All individuals were recruited from the Singapore General Hospital in Singapore. The diagnosis of PD was based on established criteria [15]. After approval from Hospital Scientific Committee and informed consent, blood samples were drawn for DNA extraction from patients and controls.

### Genetic Analysis

All individuals were genotyped by using a matrix-assisted laser desorption/ionization time-of-flight (MALDI-TOF) mass spectrometry on a MassArray system (Sequenom, San Diego, USA). Briefly, locus-specific polymerase chain reaction (PCR) and detection primers were designed using the MassArray Assay Design 3.0 software (Sequenom, San Diego, USA). The sample DNAs were amplified by primers flanking the targeted sequence, followed by dephosphorylation and allele-specific primer extension. Cleaned extension products were loaded into a 384-format Spectro-Chip, and subjected to MALDI-TOF mass spectrometry. The resultant data were analyzed by the Sequenom MassArray Typer software (Sequenom, San Diego, USA).

### Statistical Analysis

We assessed Hardy-Weinberg equilibrium (HWE) in cases and controls with a Fisher’s exact test. The frequencies of the alleles and genotypes in the patients and control groups were analyzed using the Chi-square test. A two-tailed P-value ≤ 0.05 was considered statistically significant. Statistical analysis was performed using the Statistical Package for the Social Sciences version 16.0 (SPSS, Chicago, IL, USA) for Windows.

## Results

Data from a total of 2342 subjects including 1200 PD patients (810 from mainland China, 390 from Singapore) and 1142 normal controls (750 from mainland China, 392 from Singapore) were analyzed. All polymorphisms were in Hardy-Weinberg equilibrium for PD patients and controls in both the mainland China and Singapore series. The characterizations of the studied populations are presented in Table S1. Distributions of genotype and minor allele frequencies are presented in Table S2-4.

In the mainland China cohort, the mean age at onset of PD symptoms was 53.9±10.92 years. The minor allele frequencies (MAF) of rs4880 were similar in both PD patients and controls (OR = 0.096, 95% CI: 0.737–1.114, P = 0.349), as well as the other three SNPs in SYN III (rs3788470: OR = 0.923, 95% CI: 0.792–1.074, P = 0.300; rs3827336: OR = 0.956, 95% CI: 0.799–1.143, P = 0.622; rs5998557: OR = 0.928, 95% CI: 0.795–0.083, P = 0.341). See more detailed datas in Table S2.

In the Singapore cohort, the mean age at onset of PD symptoms was 59.48±17.08 years. There were also no significant differences in the minor allele frequencies (MnSOD rs4880: OR = 0.872, 95% CI: 0.657–1.158, P = 0.343; SYN III rs3788470: OR = 1.038, 95% CI: 0.839–1.284, P = 0.733; rs3827336: OR = 0.979, 95% CI: 0.754–1.272, P = 0.875; rs5998557: OR = 1.029, 95% CI: 0.832–1.275, P = 0.790). More specific datas are displayed in Table S3.

A pooled analysis of our two Asian cohorts also did not reveal any significant differences in the distribution of genotype polymorphisms of MnSOD and SYN III between PD and controls (Table S4).

## Discussion

Oxidative stress (OS) is a primary pathogenic mechanism of nigral dopaminergic (DA) cell death in PD [16]. The nigrostriatal system is particularly susceptible to toxin-based insults. Although selective uptake of toxic metabolites may account in part for this sensitivity, recent discoveries indicate that systemic mitochondrial complex I inhibition also causes selective nigrostriatal toxicity implicating the intrinsic vulnerability of this system to toxic insults [17]. Mitochondrial dysfunction may provide a unifying genetic and pathophysiological explanation for AD, PD, and other neurodegenerative diseases. The normal function of mitochondria could be affected by the accumulation of reactive oxygen species (ROS), which is caused by the oxidative damage and decreased activity of MnSOD result from gene mutation. MnSOD, an endogenous anti-oxidant enzyme, is immunocytochemically detectable in brain regions which are prone to ischemic injury and oxidative stress such as the hippocampal formation, the cerebral cortex and the substantia nigra [18], [19]. Polymorphisms of MnSOD gene may influence the onset risks of brain tumor, Alzheimer’s disease and some other nological disorders [20], [21]. Recent research by Fong et al revealed an increased risk of PD among Taiwanese C allele carriers of MnSOD with history of pesticide exposure [3]. Additionally, Shimoda have reported the Mn-superoxide dismutase (MnSOD) C allele is significantly associated with familial PD in Japanese patients in 1997 [4]. In agreement with two Genome-wide association studies and other three European studies published so far [5]–[9], our current study failed to replicate the association between MnSOD and PD.

As a member of the synapsins, a group of neuron-specific phosphoproteins that are associated with synaptic vesicles [22], *SYN3* is located in the chromosome 22q12-q13. It has been implicated in the regulation of neurotransmitter release, synaptogenesis, and rate of axonal growth and size of the growth cones of developing neurons [23], [24]. In addition, SYN3 regulates the dopaminergic neurotransmission [25]. The association of polymorphism of SYN3 with multiple sclerosis and several neuropsychiatric diseases have been reported [10]–[12], [22]–[24]. With a view to the peculiar function of *SYN3*, we attempted to explore the relationship between the gene polymorphisms and the pathogenesis of PD with a large case-control study. Neither the separate data sets nor the merged data show any significant differences between PD patients and controls in the genotype and allele frequency distributions of three *SNPs* (rs3788470, rs3827336, rs5998557) of the *SYN3 gene*.

Several potential reasons may account for the divergent findings across different studies. First, genetic heterogeneity among different populations and population stratification differences between cases and controls may lead to different results. Second, differences in sample size and methodology must be taken into the interpretation. Third, gene–environment and gene–gene interactions may be involved in the development of PD. The four SNPs could also interact with other variants and some environmental factors, which may account for the inconsistent result.

In conclusion, our results indicate that genetic variations in *MnSOD* and *SYN3* do not influence the risk of developing PD among the two Chinese populations (one from mainland China and one from Singapore). However, further studies of these and other SNPs within the MnSOD gene and SYN3 gene in ethnic Chinese in other countries may be useful to validate our finding.

## Supporting Information

Table S1 **The demographic data of the studied populations.**
(DOCX)

Table S2 **Distribution of genotype polymorphisms of MnSOD and SYN III Among Parkinson’s Disease (PD) and Controls in Mainland China.**
(DOCX)

Table S3 **Distribution of genotype polymorphisms of MnSOD and SYN III Among Parkinson’s Disease (PD) and Controls in Singapore.**
(DOCX)

Table S4 **Distribution of genotype polymorphisms of MnSOD and SYN III Among Parkinson’s Disease (PD) and Controls in the Merged Population.**
(DOCX)
