# Supplementary material for: No Association of Four Candidate Genetic Variants in MnSOD and SYNIII with Parkinson's Disease in Two Chinese Populations
Source: PLoS One. 2014 Feb 26;9(2):e88050. doi: 10.1371/journal.pone.0088050 (PMC3935830; doi:10.1371/journal.pone.0088050)
Supplement: Table S1 — The demographic data of the studied populations. (DOCX) [file pone.0088050.s001.docx]

**TABLE S1. The demographic data of the studied populations**

|  | Mainland China | | Singapore | |
| --- | --- | --- | --- | --- |
|  | PD (n=810) | Controls (n=750) | PD(n=390) | Controls(n=392) |
| Age(years)^a^ | 57.83±10.78 | 55.25±12.84 | 67.12±9.20 | 58.87±8.56 |
| Female (%) | 43.3 | 46.9 | 42.8 | 46.4 |
| Age at onset (years)^a^ | 53.9±10.92 |  | 59.48±17.08 |  |

Key: a, Data are mean ± SD. PD, Parkinson’s disease.
