# Supplementary material for: No Association of Four Candidate Genetic Variants in MnSOD and SYNIII with Parkinson's Disease in Two Chinese Populations
Source: PLoS One. 2014 Feb 26;9(2):e88050. doi: 10.1371/journal.pone.0088050 (PMC3935830; doi:10.1371/journal.pone.0088050)
Supplement: Table S2 — Distribution of genotype polymorphisms of MnSOD and SYN III Among Parkinson’s Disease (PD) and Controls in Mainland China. (DOCX) [file pone.0088050.s002.docx]

**TABLE S2. Distribution of genotype polymorphisms of MnSOD and SYN III Among Parkinson’s Disease (PD) and Controls in Mainland China**

|  | PD, n (%) | Controls, n (%) | OR (95% CI) | *P-*value |
| --- | --- | --- | --- | --- |
| MnSOD |  |  |  |  |
| rs4880 genotype |  |  |  |  |
| CC | 15（2.0） | 11（1.6） |  |  |
| TC | 185（23.5） | 184（26.6） |  |  |
| TT | 586（74.5） | 497（71.8） |  | 0.375 |
| C | 215（13.7） | 206（14.9） |  |  |
| T | 1357（86.3） | 1178（85.1） | 0.906（0.737 1.114） | 0.349 |
| SYN III |  |  |  |  |
| rs3827336 genotype |  |  |  |  |
| GG | 29（3.6） | 32（4.4） |  |  |
| GC | 248（30.9） | 226（30.8） |  |  |
| CC | 525（65.5） | 475（64.8） |  | 0.753 |
| G | 306（19.1） | 290（19.8） |  |  |
| C | 1298（80.9） | 1176（80.2） | 0.956（0.799 1.143） | 0.622 |
| rs3788470 genotype |  |  |  |  |
| TT | 76（9.5） | 67（9.1） |  |  |
| GT | 337（42.0） | 341（46.2） |  |  |
| GG | 390（48.5） | 330（44.7） |  | 0.240 |
| T | 489（30.4） | 475（32.2） |  |  |
| G | 1117（69.6） | 1001（67.8） | 0.923（0.792 1.074） | 0.300 |
| rs5998557 genotype |  |  |  |  |
| CC | 78（9.8） | 63（8.9） |  |  |
| GC | 328（41.2） | 328（46.2） |  |  |
| GG | 391（49.0） | 319（44.9） |  | 0.143 |
| C | 484（30.4） | 454（32.0） |  |  |
| G | 1110（69.6） | 966（68.0） | 0.928（0.795 0.083） | 0.341 |

Key: PD, Parkinson’s disease; SNP, single nucleotide polymorphism; OR, odds ratio; CI, conﬁdence interval.
