# Supplementary material for: No Association of Four Candidate Genetic Variants in MnSOD and SYNIII with Parkinson's Disease in Two Chinese Populations
Source: PLoS One. 2014 Feb 26;9(2):e88050. doi: 10.1371/journal.pone.0088050 (PMC3935830; doi:10.1371/journal.pone.0088050)
Supplement: Table S3 — Distribution of genotype polymorphisms of MnSOD and SYN III Among Parkinson’s Disease (PD) and Controls in Singapore. (DOCX) [file pone.0088050.s003.docx]

**TABLE S3. Distribution of genotype polymorphisms of MnSOD and SYN III Among Parkinson’s Disease (PD) and Controls in Singapore**

|  | PD, n (%) | Controls, n (%) | OR (95% CI) | *P-*value |
| --- | --- | --- | --- | --- |
| MnSOD |  |  |  |  |
| rs4880 genotype |  |  |  |  |
| CC | 8（2.1） | 6（1.5） |  |  |
| TC | 89（23.0） | 107（27.4） |  |  |
| TT | 290（74.9） | 277（71.1） |  | 0.329 |
| C | 105（13.6） | 119（15.3） |  |  |
| T | 669（86.4） | 661（84.7） | 0.872（0.657 1.158） | 0.343 |
| SYN III |  |  |  |  |
| rs3827336 genotype |  |  |  |  |
| GG | 12（3.1） | 8（2.1） |  |  |
| GC | 111（28.5） | 121（31.2） |  |  |
| CC | 266（68.4） | 259（66.7） |  | 0.516 |
| G | 135（17.4） | 137（17.7） |  |  |
| C | 643（82.6） | 639（82.3） | 0.979（0.754 1.272） | 0.875 |
| rs3788470 genotype |  |  |  |  |
| TT | 46（11.8） | 37（9.5） |  |  |
| GT | 159（40.9） | 172（44.0） |  |  |
| GG | 184（47.3） | 182（46.5） |  | 0．474 |
| T | 251（32.3） | 246（31.5） |  |  |
| G | 527（67.7） | 536（68.5） | 1.038（0.839 1.284） | 0.733 |
| rs5998557 genotype |  |  |  |  |
| CC | 37（9.6） | 37（9.5） |  |  |
| GC | 174（44.8） | 171（43.7） |  |  |
| GG | 177（45.6） | 183（46.8） |  | 0.944 |
| C | 248（32.0） | 245（31.3） |  |  |
| G | 528（68.0） | 537（68.7） | 1.029（0.832 1.275） | 0.790 |

Key: PD, Parkinson’s disease; SNP, single nucleotide polymorphism; OR, odds ratio; CI, conﬁdence interval.
