# Supplementary material for: No Association of Four Candidate Genetic Variants in MnSOD and SYNIII with Parkinson's Disease in Two Chinese Populations
Source: PLoS One. 2014 Feb 26;9(2):e88050. doi: 10.1371/journal.pone.0088050 (PMC3935830; doi:10.1371/journal.pone.0088050)
Supplement: Table S4 — Distribution of genotype polymorphisms of MnSOD and SYN III Among Parkinson’s Disease (PD) and Controls in the Merged Population. (DOCX) [file pone.0088050.s004.docx]

**TABLE S4. Distribution of genotype polymorphisms of MnSOD and SYN III Among Parkinson’s Disease (PD) and Controls in the Merged Population**

|  | PD, n (%) | Controls, n (%) | OR (95% CI) | *P-*value |
| --- | --- | --- | --- | --- |
| MnSOD |  |  |  |  |
| rs4880 genotype |  |  |  |  |
| CC | 23（1.9） | 17（1.6） |  |  |
| TC | 274（23.4） | 291（26.9） |  |  |
| TT | 876（74.7） | 774（71.5） |  | 0.132 |
| C | 320（13.6） | 325（15.0） |  |  |
| T | 2026（86.4） | 1839（85.0） | 0.894（0.756 1.056） | 0.187 |
| SYN III |  |  |  |  |
| rs3827336 genotype |  |  |  |  |
| GG | 41（3.4） | 40（3.6） |  |  |
| GC | 359（30.1） | 347（31.0） |  |  |
| CC | 791（66.4） | 734（65.4） |  | 0.892 |
| G | 441（18.5） | 427（19.0） |  |  |
| C | 1941（81.5） | 1815（81.0） | 0.966（0.833 1.119） | 0.644 |
| rs3788470 genotype |  |  |  |  |
| TT | 122（10.2） | 104（9.3） |  |  |
| GT | 496（41.6） | 513（45.4） |  |  |
| GG | 574（48.2） | 512（45.3） |  | 0.169 |
| T | 740（31.0） | 721（31.9） |  |  |
| G | 1644（69.0） | 1537（68.1） | 0.960（0.848 1.086） | 0.514 |
| rs5998557 genotype |  |  |  |  |
| CC | 115（9.7） | 100（9.1） |  |  |
| GC | 502（42.4） | 499（45.3） |  |  |
| GG | 568（47.9） | 502（45.6） |  | 0.360 |
| C | 732（30.9） | 699（31.7） |  |  |
| G | 1638（69.1） | 1503（68.3） | 0.961（0.848 1.089） | 0.532 |

Key: PD, Parkinson’s disease; SNP, single nucleotide polymorphism; OR, odds ratio; CI, conﬁdence interval.
